# How People Use Social Information to Find out What to Want in the Paradigmatic Case of Inter-temporal Preferences

**DOI:** 10.1371/journal.pcbi.1004965

**Published:** 2016-07-22

**Authors:** Michael Moutoussis, Raymond J. Dolan, Peter Dayan

**Affiliations:** 1 Wellcome Trust Centre for Neuroimaging, University College London, London, United Kingdom; 2 Max Planck–UCL Centre for Computational Psychiatry and Ageing Research, University College London, London, United Kingdom; 3 Gatsby Computational Neuroscience Unit, University College London, London, United Kingdom; Brain and Spine Institute (ICM), FRANCE

## Abstract

The weight with which a specific outcome feature contributes to preference quantifies a person’s ‘taste’ for that feature. However, far from being fixed personality characteristics, tastes are plastic. They tend to align, for example, with those of others even if such conformity is not rewarded. We hypothesised that people can be uncertain about their tastes. Personal tastes are therefore uncertain beliefs. People can thus learn about them by considering evidence, such as the preferences of relevant others, and then performing Bayesian updating. If a person’s choice variability reflects uncertainty, as in random-preference models, then a signature of Bayesian updating is that the degree of taste change should correlate with that person’s choice variability. Temporal discounting coefficients are an important example of taste–for patience. These coefficients quantify impulsivity, have good psychometric properties and can change upon observing others’ choices. We examined discounting preferences in a novel, large community study of 14–24 year olds. We assessed discounting behaviour, including decision variability, before and after participants observed another person’s choices. We found good evidence for taste uncertainty and for Bayesian taste updating. First, participants displayed decision variability which was better accounted for by a random-taste than by a response-noise model. Second, apparent taste shifts were well described by a Bayesian model taking into account taste uncertainty and the relevance of social information. Our findings have important neuroscientific, clinical and developmental significance.

## Author Summary

People often change their preferences in the light of what others choose. One form of such change is ‘epistemic trust’ for preferences, i.e. preference alignment over and above any direct benefits that may accrue. We sought to explain preference shifting in terms of normative Bayesian inference in which, along with updating beliefs about what the world is like, and what the correct or profitable answers are given one's tastes, subjects also learn about their own personal tastes when these are incompletely certain. In a novel study based on a well-established paradigms, 740 young people expressed their tastes about the degree to which they preferred a smaller but immediate, versus a larger but delayed, reward. They did this both before and after learning about another agent’s choices. We found taste changed between the two assessments to a degree that was correlated with subjects’ choice variability in the absence of social influence. This is consistent with our Bayesian model if, for instance, people make choices by taking random samples from their own uncertain beliefs. Younger people were influenced by others more than older ones, and this observation was explained in the model by the former being less certain about their own preferences.

## Introduction

People change their choices, usually in the direction of conformity, when they learn what others value [1]. Reasons for this include the mechanistic, such as forms of priming; the instrumental, such as avoiding the dangers of social non-conformity or to seek social approval; and the epistemic, in which people who are unsure about their own preferences use observations of those of others as data. Interpersonal influence, such as choice convergence, has been extensively studied in instrumental settings. First, alignment with others is explicitly sought when conformity is itself rewarded [2]. Second, choices converge if conformity is not rewarded but choices result explicitly from shared information about the state of the world [3,4]. Toelch and Dolan [1] termed these (social-)normative and informational influence respectively. In contrast, here we focus on epistemic preference change where there is no explicit calculation of improved outcomes [5,6] (though this effect may have even contributed to some behaviour change during experiments that examined instrumental conformity).

In this study we use the term ‘taste’ in a strict sense to mean the function directly mapping stimulus attributes to utility [7]. As an example, if I used to choose oranges over apples but, having gathered social information, I now choose apples because I explicitly estimate that oranges don’t sell [4], this is not a preference change in the sense of ‘taste’. Versions of preference (taste) change have been observed in domains as diverse as oenophilia [8] and pain [9], though more typically in contexts where the values of others have to be inferred indirectly from what amounts to price-lists provided in the experiments. Unlike the present work these studies have not examined the computational structure of such changes. Here, we sought to examine epistemic preference change occasioned by the demands of learning about other’s choices [10].

A important domain in which such effects have been shown is temporal discounting [11], which quantifies the extent to which a person prefers a temporally proximal reward over a distal one, even if the latter is larger. Discounting is of economic [11] and psychiatric [11–14] importance. Thus understanding how social influences might lead people to develop or repair maladaptive discounting is of special clinical relevance. By contrast with many other domains of preference, discounting also enjoys extensively tested mathematical formalizations.

In a recent study [11], we showed that when subjects learned to make discounting choices for other individuals, their own tastes apparently changed to become more like those of these partners. Here, we sought to examine a potentially Bayesian basis for this, testing our ideas on a substantial new sample of subjects whose basic discounting preferences and demographics we also present here.

The premise for our account is that subjects are uncertain about their own taste for discounting. This is entirely plausible in the light of the substantial debate as to the rationale for discounting in the first place, as well as of how taste uncertainty may affect other domains of choice [12,13]. We thus proceed in four steps: (1) subjects’ uncertainty would be reflected in the variability of their choices, even in the absence of perturbing influences; (2) the more uncertain subjects are about their preferences, the more they would shift on learning about others; (3) this degree of preference-shifting could be described in terms of relevance, which we operationalise as the width of the distribution of preferences in a notional reference group of people to whom both the index person and the social influencer belong and (4) these effects would dominate over more complex social motives, such as those stemming from mere participation in the experiment (and thus be independent of the direction of social influence), oppositional traits (shifting away from the Other) or competitive traits ('overtaking' the other). We justify and elaborate these steps using theory and experiment.

## Methods

### Sample

In a novel study, participants were recruited from North London and Cambridgeshire as part of the Neuroscience in Psychiatry Network (NSPN). They, or their legal guardians if younger than 16, gave informed consent. The study was approved by the Cambridge Central Research Ethics Committee (12/EE/0250). We invited participants so that the final sample was equally distributed between the two genders and between the ages of 14 to 24. Participants were excluded if they currently received help for a mental health issue, if they had moderate or severe learning disability or serious neurological disorders.

### Task

We used the 'Delegated Interpersonal Discounting (DID)' task [11,14]. The task was delivered as part of a battery administered to equal numbers of male and female community dwellers between the ages of 14 and 24 in Cambridgeshire and London, as part of the Neuroscience in Psychiatry Network (NSPN) project. At the time of this study 750 participants had been recruited; 5 withdrew consent; in 4 cases, the research assistant conducting the experiment decided not to complete the task for the sake of the wellbeing of the participant (e.g. tired, unhappy). In a further 3 cases, technical problems rendered the data unusable. We therefore present the analysis of 738 cases.

The task involved three phases. In phase 1, subjects made a series of temporal discounting decisions that we used to estimate their initial value *K*_1_ in a standard hyperbolic discounting model. The index 1 stands for phase 1 of the experiment, before learning about another individual. According to this model, the value of a reward *R*_*D*_ given after a delay *D* is

$$V_{D}=R_{D}/(1+KD)$$ (1)

where *K* is the hyperbolic discounting parameter [15–17].

In phase 2, they learned to make choices expressed by another, simulated, participant whose *K* = *K*_o_ differed from theirs. Finally, in phase 3, they made more choices for themselves and the other, allowing us to assess whether their *K*_3_ ≠ *K*_1_ had changed (3 here indexes phase 3, after exposure to the partner). The *K*_o_ of the simulated participant was set to be systematically larger or smaller than *K*_1_ by a modest amount in order to provide the temptation to change.

In detail, we approximated the behaviour of participants and simulated the ‘other’ using hyperbolic value discounting followed by a softmax rule:

$$\begin{array}{l} Q_{0}=R_{0}\\ Q_{D}=\frac{R_{D}}{1+KD}\\ \pi_{D}=\frac{1}{1+e^{\frac{Q_{0}-Q_{D}}{T}}} \end{array}$$ (2)

where *π*_*D*_ is the policy probability for choosing the delayed option, *Q*_0_,*Q*_*D*_ are the action values for choosing the immediate or the delayed option (of values *R*_0_, *R*_*D*_) respectively, and *T* is the motivational currency or softmax temperature that quantifies how much a unit change in objective outcomes affects choice probability. In assessing *K*_1_ during the experiment, in order to determine *K*_*o*_ and realize the other’s choices, we made the assumption that *T* = 1, since previous work [11] with this method suggested that this would suffice. However the results below are based on fitting *T* too.

The 60 trials of phase 1 comprised 30 from a standard set covering a wide range of values of *K*, and an interleaved set of 30 from an adaptive algorithm. The latter calculated a probability distribution over the possible values of *K*_*b*_ characterising the participant under Eq 2; and then chose a pair of options likely to reduce the uncertainty (entropy) of that distribution as much as possible.

In phase 2, we chose *K*_*o*_ based on *K*_1_. Previous results [11] and pilot data led us to expect that the population would have an approximately normal distribution of ln(*K*) with a mean of roughly *μ* = −4.5 and a standard deviation of roughly *σ* = 2.3. We therefore chose *k*_*o*_ = ln(*K*_*o*_) (using lower case *k* = ln(*K*)) to be shifted from *k*_*b*_ by one *σ* either towards or away from −4.5 with probabilities 2/3 and 1/3 respectively, simulating real-life encounters that were on the whole not unlikely. We presented participants with options much like the ones in phase 1, but now asked participants “What would [name] choose?” [name] was gender-matched to the participant and likely to be encountered among their peers. It was chosen from a selection of typical names given to children born in England in the last 20 years. Once the participant made their choice *on behalf of the seeming Other*, we simulated the other's choice (using *T* = 1) and gave the participant veridical feedback as to whether or not they were correct. We presented trials until either the participant got 8 correct answers out of the most recent 10, or 60 learning trials were completed.

In phase 3, we interleaved mini-blocks of 10 trials 'choose for self', which were as in phase 1, and 10 trials 'choose for other', which were as in phase 2. We instructed participants that one of the 'choose for self' trials from the entire task would be chosen at random and the choice they made paid out for real at the appropriate delay. Participants were instructed that the task was about their “true preferences” and there was no financial incentive to make correct choices in the 'choose for other' trials.

The task was thus very similar to that used by Nicolle and co-workers [14], but optimized for delivering to large community samples. We relied on the experimental design but also in the control experiments performed by Garvert and co-workers [11] to guard against explicit instrumental explanations as well as against simple forms of priming accounting for the change (See SI of [11]). For example we made it very clear to the participants that they would be paid according to the preferences they expressed about themselves only; and that there was no “right or wrong answer” regarding what they chose for themselves. Indeed we were “interested in their own preferences”. The task was coded in MATLAB 2012a running on 12' screen laptops with the Cogent graphics toolbox (see Acknowledgments).

### Models

We first consider how to model choice variability along with modal preference, as this will play a key role in understanding preference shift. If we faced a participant with just a single delayed option and found that they chose it, say, 60% of the time, we would not be able to tell if this was because of a relatively high variability parameter (T) or because of relatively weak modal preference (K). However over many trials we used a range of triads of R_0_ and R_D_ and D to disambiguate the two parameters. In Eq 2 for example this is possible as *K* only affects the components of the delayed choice whereas *T* affects both (see also supporting information S1 Text and S1 Data).

#### Preference-temperature (KT) model

We first fitted the model of Eq 2, this time with *T* being a free parameter, to phase 1 of the experiment. We refer to this as the KT model. The maximum-a-posteriori (MAP with flat priors) fit for *k*, *T* according to this are called *k*_*b*_,*T*_*b*_

#### Preference-uncertainty (KU) model

A recently popular way of parameterizing variability is to consider subjects as sampling a value of *k* from a distribution, with choices being made according to a deterministic version of Eq 2, i.e. *T* → 0. It is natural to consider a normal distribution for *k* (i.e., a log-normal distribution for *K*):

p(k)=N(k;m,u) (3)

Subjects will choose the delayed option if *k* < ln[(*R*_*D*_ / *R*_0_−1) / *D*]; the probability of this occurring under a single sample from the distribution of Eq 3 is

$$\pi_{D}=\Psi (\ln[(R_{D}/R_{0}-1)/D];m,u)$$ (4)

where Ψ denotes the cumulative density of the normal distribution.

If the distribution of Eq (3) reflects the beliefs of the subject about *k*, then this model can be seen as using a form of matching to equate the uncertainty of beliefs with the variability of behaviour. This can also be seen as a form of random preference model [18,19], which maintains that at any given trial agents are uncertain about their exact preference for different options. Hence they draw preferences probabilistically, giving rise to variable behaviour and also to the possibility of learning from others. It is consistent with the recent emphasis on sampling in optimal decisions [20–22]. This contrasts with the KT model, where decision noise is independent from the preference between options (Eq 2). The latter is the value difference *Q*_0_−*Q*_*D*_, well-known to the agent. The KT model is in that sense a ‘trembling hand’ model where an error rate dilutes preferences [23–25].

One characteristic of the log normal distribution implied by Eq 3 is its scalar property relative to *K* (rather than *k*), in that, for a fixed standard deviation *u*, the larger *m*, the larger the standard deviation of *K*, and hence the more variable the temporal discounting behaviour associated with samples (with the additional proviso that the indifference point of the options faced by the agent remains in a similar relationship to the increasing m; we use this at a population level to explain observations about temporal preferences in the S1 Text). We use the subscripts 1 (and if necessary 3) when *m* and *u* are fitted to separate phases of the experiment. However we use *s*, *o* in the context of the preference-shift model as will be explained below. Note that the KU model is Fechnerian in form [26] as it compares the log of a stimulus attribute, ln[(*R*_*D*_ / *R*_0_−1) / *D*] to a criterion ln *K* subject to the noise *u* of Eq 4. It is only when the issue of inference over preferences arises that our (random- preference) attribution of variability to the preference term comes into its own, as we shall now see.

#### Uncertainty-relevance model of preference learning

Under the KU model, subjects have an explicit belief distribution over their temporal preferences (given in Eq 3). If they are not certain about *k*, and if they think that the ‘Other’ comes from a reference population that bears on their own possible preferences, then they may update *m*,*u* in the light of what they learn. We shall use subscript *r* for this reference population to which the self will refer. Note that unlike much other work in the Bayesian inference literature, inference here is not about the state of the world, e.g. the type of the Other [27,28], or about whether to conform or not to avoid costs [2], but rather about the tastes (i.e., preferences) of the self.

The probabilistic assumptions that underpin this account go as follows. The agent uses a reference population distribution $N(k_{r},\sigma_{r}^{2})$ to describe the likely similarity between their own preferences and that of the ‘other’. They thus consider both their own, and the other's, true values *k*_*s*_,*k*_*o*_ to be drawn from this with independent Gaussian noise with variance $\sigma_{r}^{2}$. They are assumed to know $\sigma_{r}^{2}$, since this characterizes how well the reference distribution captures them and the 'other'. However, they do not know *k*_*r*_ (and will thus integrate it out, assuming a flat prior).

Although *k*_*s*_ is their true temporal discounting preference, participants are uncertain about it. We model this uncertainty by saying that the subjects have information *d*_*s*_ about *k*_*s*_ equivalent to a normal likelihood distribution $p(d_{s};k_{s})\propto \exp(-{\left(k_{s}-{\hat{k}}_{s}(d_{s})\right)}^{2}/2{\hat{\sigma }}_{s}^{2}(d_{s}))$, where ${\hat{k}}_{s}(d_{s})$ is the mode of the likelihood and ${\hat{\sigma }}^{}{}_{s}(d_{s})$ the width. Similarly, the choices *d*_*o*_ of the ‘other’ license inference about their true preferences as $p(d_{o};k_{o})\propto \exp(-{\left(k_{o}-{\hat{k}}_{o}(d_{o})\right)}^{2}/2{\hat{\sigma }}_{o}^{2}(d_{o}))$, where ${\hat{k}}_{o}(d_{o})$ is the mode of the likelihood, and ${\hat{\sigma }}^{}{}_{o}(d_{o})$ its width.

Putting these probabilistic facts together, we find that *d*_*o*_ provides information about *k*_*r*_; which then provides information about *k*_*s*_ as a prior. More formally,

$$\begin{array}{l} p(k_{s}|d_{s},d_{o})=\int_{k_{r},k_{o}}p(k_{s},k_{r},k_{o}|d_{s},d_{o})dk_{o}dk_{r}\\ \propto \int_{k_{r},k_{o}}p(d_{s},d_{o}|k_{s},k_{r},k_{o})p(k_{s},k_{r},k_{o})dk_{o}dk_{r}\\ \propto p(d_{s};k_{s})\int_{k_{r},k_{o}}p(d_{o};k_{o})p(k_{s},k_{o}|k_{r})p(k_{r})dk_{o}dk_{r}\\ \propto N(k_{s};{\hat{k}}_{s}(d_{s}),{\hat{\sigma }}_{s}^{2}(d_{s}))\int_{k_{r},k_{o}}N(k_{o};{\hat{k}}_{o}(d_{o}),{\hat{\sigma }}_{s}^{2}(d_{o}))p(k_{s}|k_{r})p(k_{o}|k_{r})dk_{o}dk_{r}\\ =N(k_{s};{\hat{k}}_{s}(d_{s}),{\hat{\sigma }}_{s}^{2}(d_{s}))N(k_{s};{\hat{k}}_{o}(d_{o}),2\sigma_{r}^{2}+{\hat{\sigma }}_{o}^{2}(d_{o})) \end{array}$$ (5)

Where the last step was obtained through convolving the three Gaussian terms to be integrated by completing the square of the product exponent. The resulting product of Gaussians is also Gaussian with variance $\sigma_{s}^{2}={\left({\hat{\sigma }}_{s}^{-2}(d_{s})+{\left(2\sigma_{r}^{2}+{\hat{\sigma }}_{o}^{2}(d_{o})\right)}^{-1}\right)}^{-1}$ and mean as per:

$$p(k_{s}|d_{s},d_{o})\propto N\left(k_{s};\sigma_{s}^{2}\left[{\hat{\sigma }}_{s}^{-2}(d_{s}){\hat{k}}_{s}(d_{s})+{\left(2\sigma_{r}^{2}+{\hat{\sigma }}_{o}^{2}(d_{o})\right)}^{-1}{\hat{k}}_{o}(d_{o})\right],\sigma_{s}^{2}\right)$$ (6)

**Fig 1:**
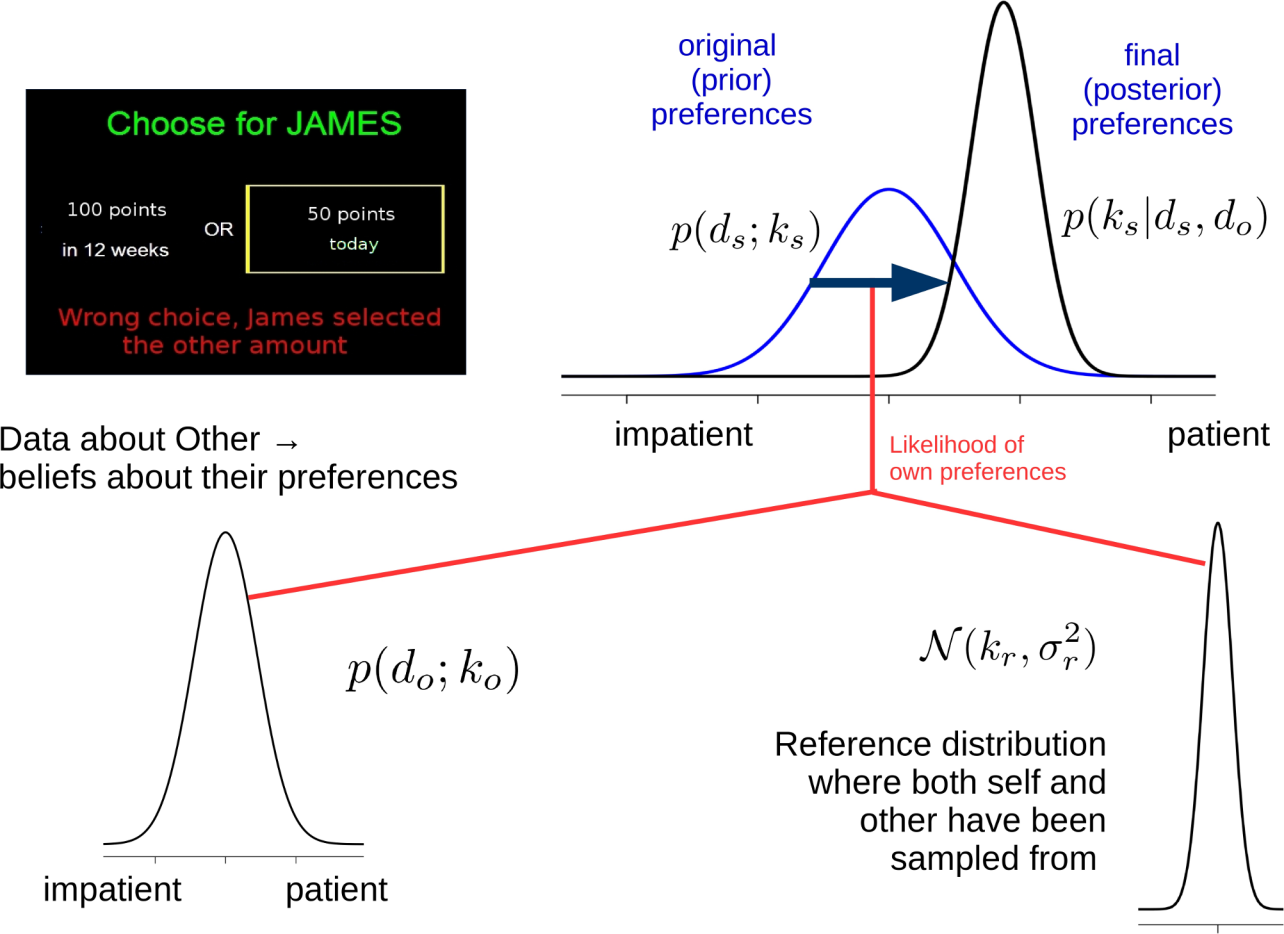
Uncertainty-relevance model of preference shift. Before information about the ‘other’ is seen, beliefs about the reference distribution are uninformative and so the original beliefs about the self are proportional to the likelihood *p*(*d*_*s*_; *k*_*s*_). Once data *d*_*o*_ about the other are seen, the likelihood of *k*_*o*_ combines with the conditional probabilities that *k*_*s*_ and *k*_*o*_ as they are drawn from the reference distribution; this combination multiplies the beliefs about the self to yield the posterior (shifted) *k*_*s*_. This is a schematic representation of Eq 5 (see e.g. its penultimate line).

The structure of probabilistic inference in the preference-shift model is depicted in Fig 1.

Exactly the same rationale implies that a participant’s belief about *k*_*o*_ can be written using $\sigma_{o}^{2}={\left({\hat{\sigma }}_{o}^{-2}(d_{o})+{\left(2\sigma_{r}^{2}+{\hat{\sigma }}_{s}^{2}(d_{s})\right)}^{-1}\right)}^{-1}$ as

$$p(k_{o}|d_{s},d_{o})\propto N\left(k_{o};\sigma_{o}^{2}\left[{\hat{\sigma }}_{o}^{-2}(d_{o}){\hat{k}}_{o}(d_{o})+{\left(2\sigma_{r}^{2}+{\hat{\sigma }}_{s}^{2}(d_{s})\right)}^{-1}{\hat{k}}_{s}(d_{s})\right],\sigma_{o}^{2}\right)$$ (7)

We assume that Eq 6 is used to make choices for the self during phase 3; and that Eq 7 is used to make choices for the ‘other’ during phases 2 and 3. We fit the Gaussian likelihoods that enter these equations in a filtering manner, i.e. choices at trial *t* use the likelihood of all data 1…*t*-1.

There are, however two further problems to do with choice variability. First, participants are not incentivised in an explicit monetary way to make correct choices for the other; we thus consider them to arise according to Eq 4, but relaxed according to a temperature parameter

*τ*_*o*_, so that (dropping the dependency on *R*_0_,*R*_*D*_,*D*) the policy *π*_*Do*_ of choosing the delayed option on behalf of the ‘other’ is further transformed:

$$\pi_{Do}\leftarrow \frac{\pi_{Do}^{\frac{1}{\tau_{o}}}}{\pi_{Do}^{\frac{1}{\tau_{o}}}+{\left(1-\pi_{Do}\right)}^{\frac{1}{\tau_{o}}}}$$ (8)

Finally, self-choices were subject to a lapse process, implying that the true probability of taking and action (e.g. the delayed one) was

$$\pi_{D}^{\prime }=\pi_{D}(1-\xi )+\xi /2$$ (9)

We considered that the choices of the ‘other’, *d*_*o*_, might be subject to a similar lapse process. However in the event *ξ* assumed low values (median 0.015) so we considered the effect of the other’s lapse rate to the eventual choices of the participant to be negligible.

Thus the Bayesian model had 5 parameters in total: ${\hat{k}}_{s}(d_{s}),{\hat{\sigma }}_{s}^{2}(d_{s})$, characterising self-knowledge; *σ*_*r*_ determining the compactness of the reference class; and *τ*_*o*_,*ξ*, the excess other- and self-noise parameters. When it came to the PS model we fitted these 5 parameters to all the data from each participant at once. We will denote the fitted parameters ${\hat{k}}_{s}=m_{s},\sqrt{{\hat{\sigma }}_{s}^{2}}=u_{s}$ for brevity.

### Data analysis

We first fitted the classic hyperbolic model and the preference-uncertainty model to the data from phase I of the task. We found (see below) that the preference-uncertainty model was of sufficient quality to use as the backbone for the preference-shift Bayesian schema.

The mainstay of our model-fitting was Markov-Chain Monte Carlo (MCMC) with weakly informative priors and the Component-wise Hit-And-Run Metropolis algorithm, implemented in the ‘LaplacesDemon’ software package [29]. All point estimates reported here are the medians of the posterior distributions of the respective variables (once stationarity was achieved). The phase 1 data were fitted with fixed-effects models (KU and KT). A full hierarchical Bayesian, random-effects analysis of the PS model had too high a dimensionality (740x5 = 3700 parameters) to be fit using MCMC. We therefore fitted it in stages. First, we fitted each individual participant separately, using uninformative priors and a Laplace approximation to the maximum-likelihood as initial conditions–a fixed-effects approach. Second, we used the point estimates of the parameters for each participant to construct an estimate of the distribution of each parameter over our sample. To this effect, and in the first instance, we ignored a small minority of participants whose data did not constrain the model well, i.e. where the stationary distribution was not achieved within 2 million un-thinned samples and / or when the effective sample size was less than 100, indicating poor mixing. Third, following the philosophy of type-2 or empirical Bayesian maximum-likelihood fitting [30], we used our estimate of the sample distributions of the parameters as priors for re-estimating individual parameters.

## Results

### Preference-temperature vs preference-uncertainty models

We first present the analysis of phase I of the experiment, as the results crucially informed our modelling choices for all further analyses. The classic KT model yielded a distribution of preferences over the population that was close to the ones we expected. We expected a mean ln(*K*_1_) of roughly *μ* = −4.5 and a standard deviation of roughly *σ* = 2.3. We obtained -4.67 with SD = 1.82, justifying a posteriori our choice the choice of *K*_*o*_ for the simulated Other being 2.3 ln (+/-)units away from the Self in phases II and III. *T* had a mean of 1.54 (SD = 1.36).

**Fig 2:**
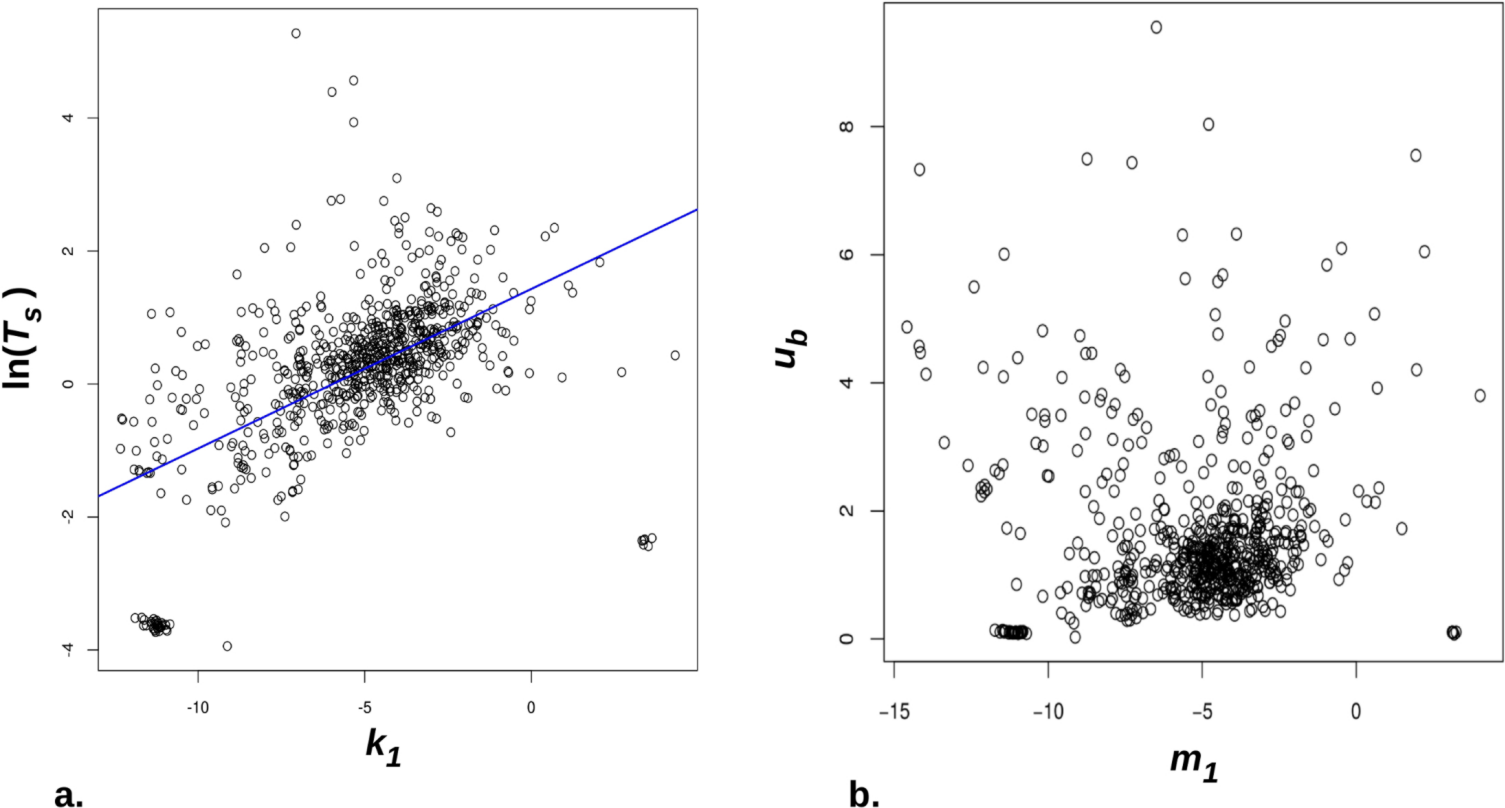
**a.** Correlation between *k*_*b*_ and *T*_*s*_ in the population. ln(*T*_*s*_) is plotted against *k*_*b*_, as the latter is already in *ln* units and the two enter Eq 2 on the same footing. Pearson *r* = 0.55, p < 1e-70. **b.** Similar plot for the KU parameterisation. *r* = -0.03, p = 0.37. Note two 'clumps' near *u*_*b*_ ~ 0 (or ln(*T*_*s*_) approx. -4 to -2) which appear separate from main cloud of points.

We unexpectedly found a powerful correlation between *K*_1_ and *T* in the population, as seen in Fig 2A. This hints that the KT formulation is problematic, as there is nothing in the constructs themselves that suggest that, for example, people who prefer not to wait should not exercise their preference as consistently as those who do wait. Such a high correlation raises the possibility that these measures of discounting and behavioural variability may influence each other, either as a neural phenomenon or an analytical artefact.

The KU formulation abolished this correlation (Fig 2B). We therefore performed model comparison to determine whether it sacrificed quality of fit to achieve this, or whether it was as good in this respect. In the event not only did the KU model capture the correlation between preference and noise in a natural manner, but it also fit the data slightly more proficiently, despite having the same number of parameters. 64% of participants had a better log-likelihood over phase 1 choices for the KU model (SEM 1.8%, Wilcoxon p = 1.7e-11, BIC difference over 738 participants = 740, mean KU log-likelihood = -23.8, mean KT LL = -24.3).

In the KU formulation, even if the mean *m*_*s*_ and variance *u*_*s*_ of ln(*K*) are uncorrelated across the population, the mean of *K* and the variance of *K* will in general be correlated. Through the sampling procedure inherent to KU this will also affect the variability in choices, although the precise nature of this effect will depend on the actual options used to probe discounting (See S1 Text). Fig 2B shows that the inferred values of *m*_*1*_ and *u*_*1*_ across the population are indeed uncorrelated. Reassuringly, *m*_*1*_ correlates closely with the inferred ln(*K*_1_) (*r* = 0.99, p < 1e-10) and *u*_*1*_ being very significantly correlated with *T*_*s*_ (and ln(*T*_*s*_); *r* (ln(*T*_*s*_), *u*_*1*_) = 0.61, p < 1e-10). The former relationship is reassuring as option pairs that are indifferent with respect to one model are also indifferent with respect to the other. The latter relationship is also reassuring in terms of face validity.

Having established KU as our preferred parametrisation, we examined the demographic distribution of discounting preferences. There was no significant dependence of *m*_*1*_ or *u*_*1*_ on gender. *m*_*1*_ declined slightly but significantly with age, Pearson *r*(*m*_*1*_, *age*) = -0.10, *p* = 0.0065. The same was true for the amount of preference shifting towards the ‘other’, with older participants shifting slightly less *r*(|*m*_*3*_ -*m*_*1*_ |, *age*) = -0.12, *p* = 0.0021.

### Uncertainty–Relevance model of preference shift

**Fig 3:**
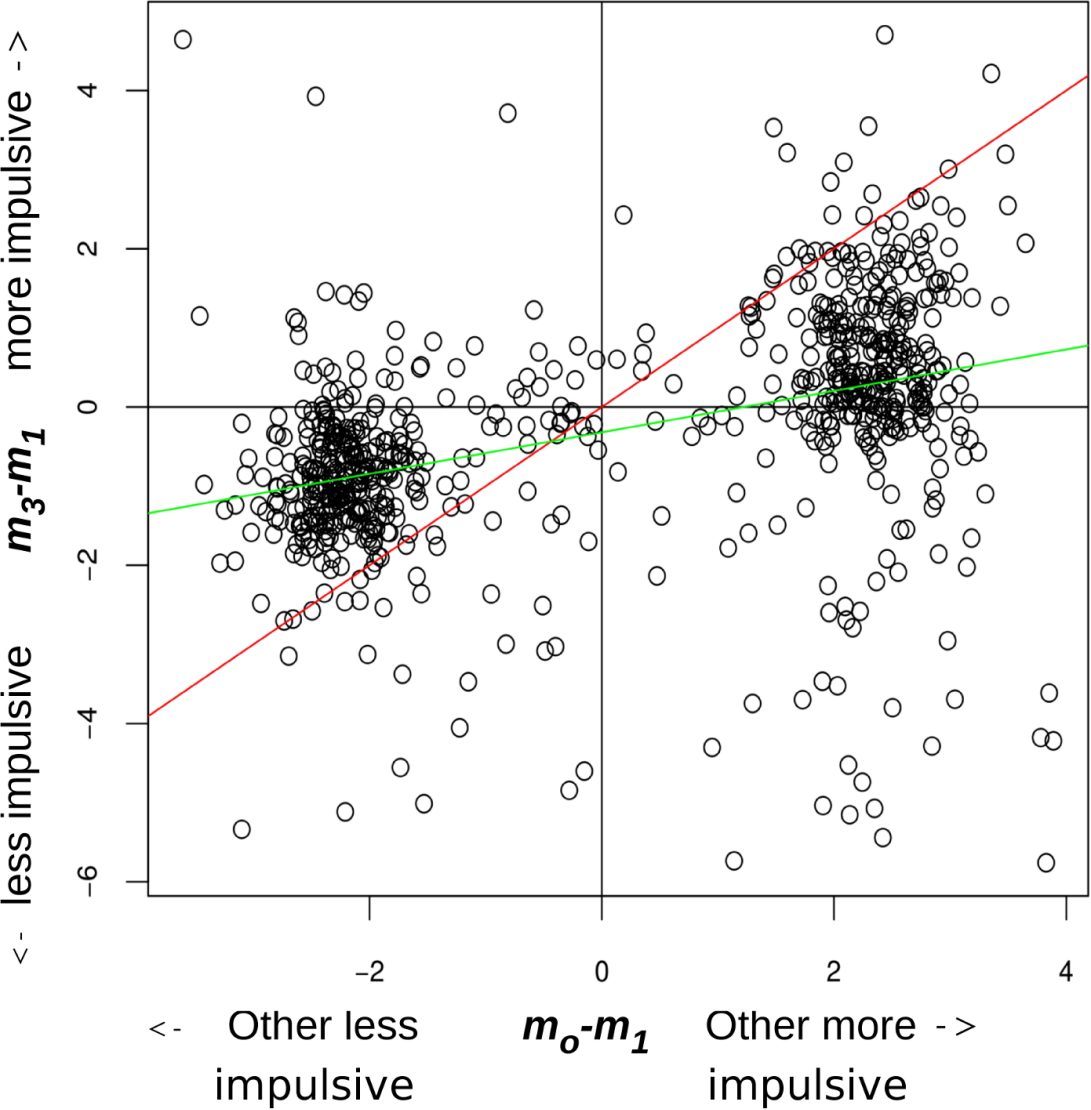
The difference between *m*-for-self after learning and before learning as a function of partner’s preference. This difference (ordinate) is plotted against the difference between *m* -for-other and *m*-for-self-before-learning. Two clusters form because we exposed participants to others that were 2.3 *ln* units away in modal preference (in either direction). Red is the identity line (fully adopting other's preference). Green is the linear regression line. It has a positive slope as expected (p ~ 0.0), but a negative intercept, denoting a slight overall bias for shifting towards more patient preferences.

Fig 3 shows how participants shifted their preferences in response to learning about the Other's preferences. The parameters plotted here are descriptive, representing the mode of the Laplace approximation to the likelihood in the pre-exposure vs. post-exposure *m*_3_−*m*_1_; versus the modal preferences of the Other-self estimated from all the choose-for-other trials. We note that the vast majority of participants shifted in the direction of the Other without overtaking them, just as the uncertainty-relevance model would predict.

**Fig 4:**
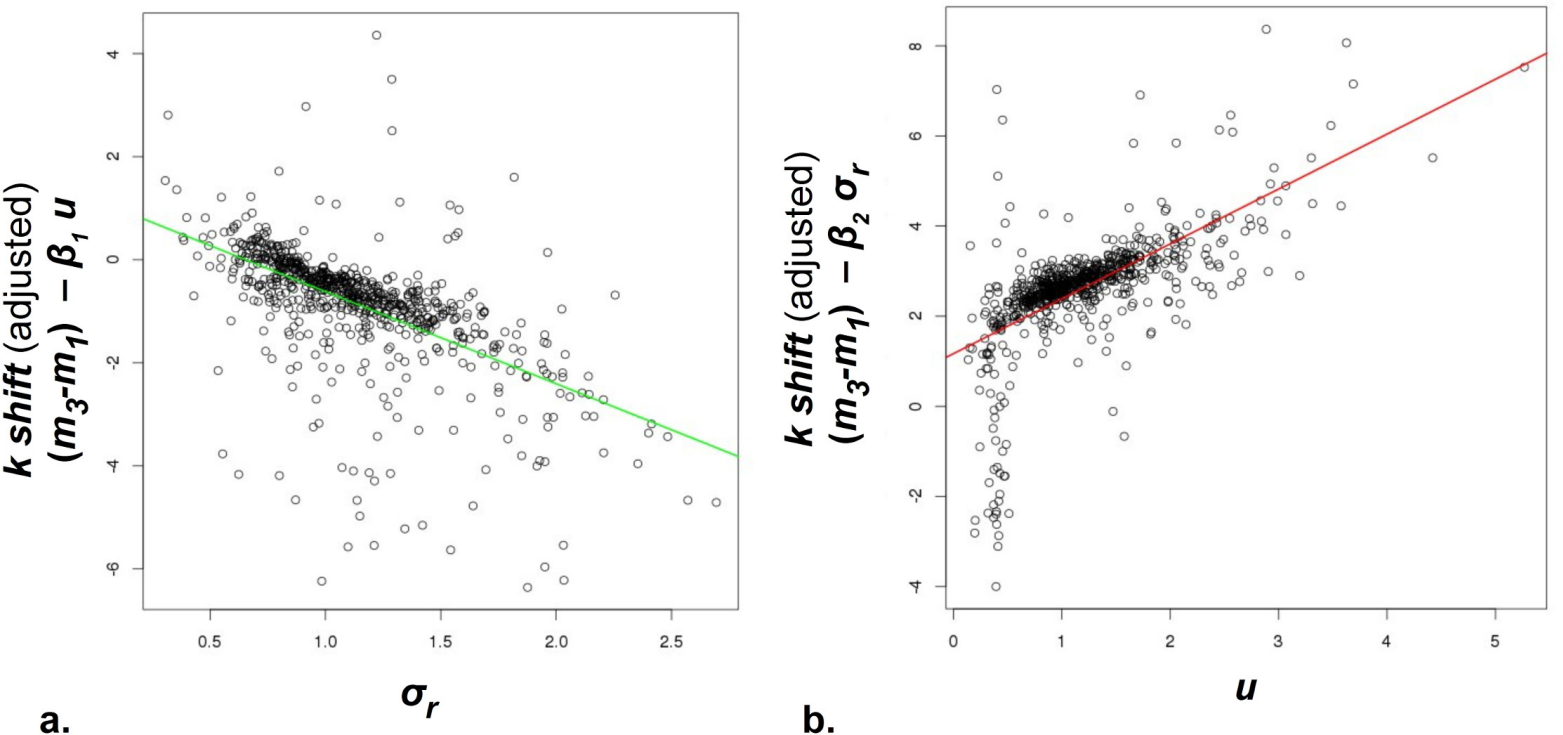
The apparent discounting shift *m*_*a*_-*m*_*b*_, considered in the direction of the ‘other’, was regressed against *σ_r_* and *u* in the whole sample, N = 738. This shift is plotted against each variable removing the variance predicted by the other. We focused on variable inter-relationships, thus ignoring y-intercept terms. **a.** Shift vs. reference dispersion *σ_r_*. The bigger the likely distance (*σ_r_*) the smaller the shift. **b.** Shift vs. preference uncertainty *u* is also in the direction predicted by Bayesian reasoning. We note that in each case the population consists of a denser core of points but also of penumbrae that slightly dilute the overall fits (coloured lines). Here we follow this more conservative whole-sample regression; see S1 Text for post-hoc quality-controlled analyses.

We then examined how the two key parameters used to describe preference-shifting in the model related to the variance in the data. We found that *σ_r_* and *u* were very significantly correlated with the shift *m*_*3*_-*m*_*1*_ over the whole sample, just as expected from the model. In terms of partial correlation coefficients, *r* (*m*_*3*_-*m*_*1*,_ *σ_r_*; *u*) = -0.56, *p <* 1e-30 while *r* (*m*_*3*_-*m*_*1*,_ *u*; *σ_r_*) = 0.61, *p <* 1e-30, and positive shifts being in the direction of the other’s discounting preference (Fig 4).

As noted for speed and convenience, we used a highly approximate procedure to estimate the *K*_*1*_ that was used as the basis of *K*_*o*_. It is possible that biases in this procedure could lead to incorrect estimates of the key parameters of the shift model (notably the fixed, low temperature T used which is not a good approximation to our final estimates). We explicitly tested for this by exploiting the fact that we randomized whether subjects were asked in phase 2 to learn about a more patient or more impulsive other. Systematic differences in the parameters between these two possibilities would imply procedural problems. There was some modest evidence for this: those who faced a more patient Other were fitted with a slightly larger *u* (mean 1.27 vs. 1.11; effect size ~ 0.24; Wilcoxon p = 0.00046) and slightly smaller *σ_r_* (mean 1.06 vs. 1.21; effect size ~ 0.44; Wilcoxon p = 5.7e-8). We were not able to establish a confound in the model that explained the slight overall bias evident in Fig 3 towards becoming more patient.

We also checked whether there were subsets of participants that shifted their preferences in a systematic way, over and above the uncertainty-relevance model. We thus allowed for an arbitrary perturbation in *k* between phases II and III of the experiment. This would allow the model to produce a high likelihood for any preference shift, as long as preferences were captured as well by the same basic discounting model (here, the KU model) but it would be agnostic as to the mechanism of this. Examples might be participants that overtake the ‘other’, or shift in the wrong direction (i.e. outside the triangles defined by the identity line and x-axis in Fig 3). We then compared the BIC values for the KU vs. perturbed models. The BIC difference in favour of the perturbation model was > 2 in 7.4% of participants and > 6 in 4.2% of participants. We therefore concluded that the overall fraction of participants where there was strong evidence for a process not captured by our main model, according to BIC conventional values, was in fact small.

Finally, we examined whether *σ_r_* or *u* explained the age-dependence of preference shifting that we observed. *σ_r_* was not significantly correlated with age but *u* declined (*r* = -0.14, *p* = 7.7e-5), and this fully mediated the decrease of preference shifting with age (shifting partial *r* for age: -0.06, p = 0.11; for *u*: -0.10, p ~ 0.0). The amount of variance in preference malleability explained by age (and mediated by *u*) in this sample was small.

## Discussion

We used the paradigmatic case of discounting to model how learning about someone else's preferences may lead to a form of learning about one's own. We tested our models in a new empirical study of over 700 young people which allowed us to make a number of novel contributions. First, we provide evidence that in the presence of social information, Bayesian reasoning updates beliefs about preferences, i.e. the personal tastes themselves, as opposed to beliefs regarding profitable decisions *given* one's tastes. Second, we show that uncertainty about one's own preferences, reflected in behavioural variability in the absence of social influence, is an important basis for a subsequent preference shift. Third, we introduce the notion of ‘reference dispersion’, which relates to epistemic trust [31,32], and which quantifies ‘how likely is it that my taste are similar to those of an other’. It is thus an estimate of similarity, and can be manipulated in future studies to provide further experimental tests of our model. The novel finding here is that 'reference dispersion' is less than the actual dispersion in the study population, quantifying how participants privilege the experimental context. Finally, we report evidence that decreasing uncertainty about one's own preferences, rather than a change in reference dispersion, accounts for a decreasing malleability in preference with increasing age.

Our study was motivated by an observation that discounting preference shifts take place even if there is no obvious, conventional, motive such as direct reward for making choices like another person's, explicit social approval, or direct gains that accrue to others. Further, the original study on which this one builds indicated that simple priming mechanisms, such as repeating previously performed choices, do not account for taste shifts [11]. Previous studies which examined taste change under social influence in domains such as preferences for facial characteristics of the opposite gender [5] addressed similar issues but did not examine their computational basis. Inferring ‘the best discounting factor for me to like’ may entail analogous distal benefits as inferring ‘the right facial characteristics for me to like’–the crucial point being that such distal benefits are not explicitly calculated but absorbed into tastes.

In our account, subjects were modelled as being uncertain about their own tastes and this uncertainty was reflected in the choices they made even before they learned about the preferences of others. We captured these characteristics in the taste-uncertainty (KU) model by assuming that subjects maintained and updated a distribution over their own taste and sampled from it to make a choice on a trial. This overall model fit the subjects’ behaviour better than the classic softmax (KT), and also explained away an otherwise surprising correlation between the hyperbolic discounting parameter *K* and the temperature *T* (see also S1 Text). Sampling matched behavioural variability to uncertainty, which is consistent with recent suggestions about the role of sampling in choice [20,33], and goes beyond the view of random preferences describing the distribution of tastes of individuals across a population, or from inevitable imperfections within a neural system [26]. The better fit of the KU model, the dependence of preference-shift on choice variability and the decrease in taste uncertainty with age suggest that choice variability substantially reflects uncertain taste rather than just ‘trembling hand’, taste-independent response noise. Uncertain taste does not by itself necessitate behavioural variability like the one we have observed. For example, people might have estimated their own modal taste (by taking many samples) and acted on that. However in real life the expression of preference uncertainty in matching behaviour may also be beneficial, somewhat analogous to that of resolving the exploration/exploitation dilemma by Thomson sampling [20,21].

Having a model that depends on beliefs about one’s own tastes renders it straightforward to see how such beliefs might normatively be influenced by evidence. However using observations of others as evidence about the self entails some interpretation. The question becomes one of epistemic trust [32], i.e., (a) deciding the extent to which the people whose choices are being observed are part of the same reference group as oneself, and, (b) whether that behaviour is indicative of their true tastes, or rather could be part of a game-theoretic interaction with inefficient or incomplete mechanism design [34]. In our simplified framework, the parameter *σ*_*r*_, the variability about the (unknown) mean of the reference population that is assumed for both self and other, captures the degree of epistemic trust; one limitation of our experiment is that we have little independent evidence about the value of *σ*_*r*_. We noted that the mean of the fitted *σ*_*r*_ = 1.13 is a little less than half the actual population dispersion for *m*_*s*,_, ~ 2.7. This could itself come from an implicit assumption by the participants that the other preferences they are learning about are of special relevance to them—an experiment-induced epistemic trust.

We also observed some asymmetry in participants' shifting, with an overall bias for shifting in a more patient direction (Fig 3, green regression line intercept). Our models accounted for this by a smaller *σ*_*r*_ (and slightly greater *u*) for those facing more patient partners. It could be that the experimental procedure exerted an influence on preferences over and above the difference between the participant's and the Other's preferences. People may have a slightly skewed belief distribution about their preferences, or perhaps a skewed sense of similarity. They may consider themselves more similar to patient people than impatient ones (perhaps because of some social stigma). Alternatively this effect may be independent of social reasoning, representing for example a slow reversion to the mean or a practice effect. In our conceptualization *σ*_*r*_ summarises all sources of relevance that influence learning and its fitting may absorb phenomena like the slight overall shift towards more patient choices. This should be understood further. We consider it important for future studies to actively manipulate interpersonal context on the basis of specific hypotheses about factors that determine epistemic trust (e.g. increased relevance induced by experimental context, out-group vs. in-group belonging) and factors best described separately (reversion to the mean, enhanced conformity to patient behaviour due to social stigma against impatience despite explicit instructions).

In such a large community sample individual variation will be more complex than our simple parametrization allowed. For example, 5.4% (40/738) of participants were fitted with very low, almost zero, taste uncertainty parameters–evident in the two clusters of points with very low *u* or *T* in Fig 2. They always chose either the larger or the sooner option. To avoid cherry-picking the data, we included all subjects in the statistical analysis. It may be, however that our options did not correctly span their temporal preferences, as they might have been either far too patient or impulsive. Equally, it is possible that, in such a large sample, they did not follow some aspects of the instructions. Most interesting is the possibility that a single preference model (here, the simple hyperbolic) is an approximation that needs to be refined by considering differences in the very structure of preferences across individuals, as beautifully suggested by Hey, Carbone and co-workers [35]. Additional analyses (SI section S3) confirmed that the Bayesian K-shift model accounted rather precisely for the majority of participants who closely followed the hyperbolic model while a further, exploratory analysis provided evidence for a different sort of uncertainty-based updating in those who do not closely adhere to hyperbolic discounting. It would be important for future research to address in more detail the variation of the structure of preference functions across individuals.

In summary, future research should dissect the nature of similarity or relevance (*σ*_*r*_) in our theory through hypothesis-based independent manipulations. In addition, individual variability of preference functions could be addressed in more detail (cf. SI section S3).

In terms of further applications, our findings suggest that other preference measures may be subject to uncertain beliefs and a similar inferential process. It would therefore be useful to have a clearer separation of the ‘taste’ vs. the ‘explicit consequence’ components of preferences in other domains; we acknowledge that this is not straightforward: for example, the issue of ‘pure time preference’ is still a matter of debate with respect to temporal discounting. One relevant domain is development, where it would be important to use longitudinal, rather than cross-sectional, studies to test our explanation that preference malleability changed with age because of increased preference certainty. There are also clinical implications–our findings suggest a mechanism by which therapeutic and malign social influence may operate. For example, clinicians use group treatments to ameliorate disorders now thought to be associated with increased discounting, especially alcohol and drug addiction. In group contexts, the presence of members that have already changed their behaviour and are close to 'graduating' is thought to be an important positive influence on new members [36]. Conversely, being a member of a group containing those with societally unfortunate preferences could lead to maladaptive contagion.

## Supporting Information

S1 Text Supporting Information for ‘How people use social information to find out what to want in the paradigmatic case of inter-temporal preferences’. (DOCX)

S1 Data R compressed file with experimental data (incl. README). (RDATA)

S1 Fig KU Model fit to the modal preference and the preference uncertainty. The fit was parametrised as per the KU model, using only trials from the phase 1 of the experiment, before exposure to the choices of another agent. A participant with fitted values near the middle of the population distribution of Fig 2 is shown. The three rows of plots represent the values of the (log) preference parameter, the (log) uncertainty parameter and the model deviance–a measure of model fit derived from the log-likelihood—as more samples are obtained from the posterior distribution of the parameters (the converged Markov Chain). The three columns show: **a.**, **d.**, **g.**: Values at consecutive thinned samples, illustrating that stability has been achieved. **b.**, **e.**, **h.**: smoothed histograms representing the posterior distributions. Note that they have very well defined peaks both for *k* and for *u*. **c.**, **f.**, **i.**: Autocorrelation plots indicating that the degree of thinning was appropriate–i.e., that consecutive samples (from the first column) used to construct the posteriors (second column) were independent.
(TIFF)

S2 Fig Scatter-plot of synthetic data. This is analogous to Fig 2A but produced by applying the experimental task to artificial agents. These agents followed the KU model with uncorrelated *km* and *ku*.
(TIFF)

S3 Fig Plots of Equation S1 for a set of agents with constant *u* and increasing *m*. The plots show the temperature parameter that an agent with the same modal discounting preference, but following the classic KT model, has to have in order to display an indistinguishable policy. **a.** The same option pair, *Ro* = 1 vs. *Rd* = 3, *D* = 10 is presented to all agents. **b.** The same Ro and D are used as in a., but *Rd* gradually increases from 2 to 4. This results in the indifference point between the options being 1 x *u* below *m*, but this is not important as long as *kind* tracks *m*.
(TIFF)

S4 Fig Preference shift plotted against explanatory parameters. This is based on fitting a linear regression *m*_*3*_ *–m*_*1*_ = *β*_*0*_ + *β*_*1*_ u +*β*_*2*_ *σ*_*r*_ + *β*_*3*_ *u σ*_*r*_ to the ‘quality-contolled’ data set only. In all plots *m*_*3*_-*m*_*1*_ is considered positive if towards the preferences of the Other, and negative in the opposite direction. **a**. Shift magnitude vs. reference population dispersion *σ*_*r*_ in the entire population. Grey: resulting regression line according to the ‘quality controlled’ dataset. **b**. Similar plot restricted to the ‘quality-controlled’ dataset. This picks out the area of high correlation in a. and excludes its penumbra. *β*_*0*_ to *β*_*3*_ are derived from this set, N = 466. **c**. preference variability is also tightly related to shift in this set, while **d.** the *u σ*_*r*_ interaction also makes a contribution, as in the simulated data. *p* for all *β* is < 1e-16.
(TIFF)

S5 Fig Evaluation of the ‘KTC’ model of discounting and Preference shift. **a.** and **b.** For about 2/3 of participants in both phases 1 and 3 the confidence interval for C includes zero (‘KTC model’: the value of the delayed option is adjusted by and individual parameter C). **c.** Comparison of BIC values for KU model vs. KTC model. The two grey lines indicated +/- 6 BIC units, conventionally taken to be ‘strong evidence’. Many more points are below these lines than above (403 vs. 108; ΔBIC = 744.9 over 648 participants in favour of KU). The KU model gives a better account of behaviour over the whole group, but there is a tail of participants where the KT+C model fits better. For most of these participants preference shifting is also better described as a change in C. **d.** Relationship between decision variability and preference shift for the 70 participants whose preference shift was best fitted by a change in C according to the KTC model. There is a very strong correlation between decision variability and shifting, as a Bayesian update would predict (*r =* 0.39, *p =* 0.00048 overall; *r =* 0.64, *p =* 3e-12 excluding the single outlier).
(TIFF)
