# Supplementary material for: How People Use Social Information to Find out What to Want in the Paradigmatic Case of Inter-temporal Preferences
Source: PLoS Comput Biol. 2016 Jul 22;12(7):e1004965. doi: 10.1371/journal.pcbi.1004965 (PMC4957786; doi:10.1371/journal.pcbi.1004965)
Supplement: S1 Text — (DOCX) [file pcbi.1004965.s001.docx]

# Supporting Information

for

How people use social information to find out what to want

# in the paradigmatic case of inter-temporal preferences

by

Michael Moutoussis PhD,^*1^ Raymond J Dolan FRS^1,3^ and Peter Dayan PhD^2^

1: Wellcome Trust Centre for Neuroimaging, University College London, 12 Queen Square,

London WC1N 3BG

* Corresponding author

Email: [m.moutoussis@ucl.ac.uk](mailto:m.moutoussis@ucl.ac.uk)

## Section S1. Identifiability of modal preference vs. decision variability

We examined whether modal preference vs. decision variability parameters were separately identifiable through our data. We found that this was indeed the case for typical participants, as is exemplified in Figure SF1.

[S1 Fig here ]

## Section S2. How correlated K and T correspond to uncorrelated m and u

In order to understand how individuals with uncorrelated *m* and *u*, as described by the KU model, might have correlated *K* and *T*, as per the softmax model, we first confirmed this in a simulation study. We first randomly chose 100 real participants that provided good parameter estimates on both KU and KT models. We checked that for this random sample, K and T were correlated as in our overall sample but *m* and *u* not significantly so. Third, we got these realistic values of *m* and *u* and fed them through the experimental task de novo, to produce artificial data. Note that this data is ignorant of K and T of the KT model and of the original trials from which the original *m* and *u* were derived. Finally, we re-fitted the KT model on the artificial data using MCMC with uninformative priors. We found that the new *K* and *T* were highly correlated in this artificial sample, Pearson r=0.55, p ~ 0. However, as in the real data, the overall procedure resulted in 15 pseudo-participants with extreme values, *ln T* > 4 or *ln T* < -4. We were concerned that these might lead to an inflated *K* – *T* correlation and excluded them. *K* and *T* remained strongly correlated in the sample excluding outliers, r=0.41, p ~ 0.0 (Figure SF1).

[ S2 Fig here ]

In order to obtain further analytical insight into this phenomenon, we asked what the equivalence temperature *Teq* of a KT agent would have to be in order to result in exactly the same policy as a KU agent when faced with one single option pair O = {*Ro*, delay=0 vs. *Rd,* delay=D > 0) . If we call *ki*(O) the indifference *k* of this option pair,

Equation S1

Now consider a set of agents all with the same *u*, but with a range of *m = ln*(K). As we noted in the main text, the normal distribution over *k* giving a log-normal distribution *K* implies an increasing variance in *K* if *m* increases but *u* is constant. In Figure SF2 we plot Eq. S1 to show that the equivalent relationship between *K* and *T* also depends on the specific options used in the task. A different relationship between obtains if the same option pair is presented to all participants, versus options whose *ki* tracks *m*. In our task we used both types of options, but the latter dominated the distribution of equivalent-temperatures. As an example we considered a representative fitted *u* of ~ 0.24 and observed that both the mean and the median of the *Teq* approximately doubled as *m* ranged from -8 to -2.

[S3 Fig here]

## Section S3. The impact of fidelity of hyperbolic discounting on the estimation of taste shift

The focus of this study was on preference shifting, rather than describing discounting itself as accurately as possible in all participants. Indeed our data (e.g. Figure 2a low-temperature ‘islands’) indicated that there were subgroups of participants that whose may not have calculated action-values in the same way as the others, and/or whose behaviour was not resolved in full detail by our experimental task. Still, we included all participants in our main analyses as this was the more conservative approach. Atypical participants like this are unlikely to yield good information about preference shifting: if our task and/or model does not precisely describe ‘where they are’, it is not so meaningful to ask ‘how much did they shift’.

We thus performed additional analyses on those participants who had well defined hyperbolic preference parameters and, given this firmer base, we asked whether the PS model captured most of the variance in their shifting in terms of its explanatory parameters. We required the peak log-likelihood for *k_1_* and *k_3_* in the choose-for-self trials, as well as the *k_o_* fitted to the participant’s behaviour in the choose-for-other trials, to be at least 3 log units above both its values at -11.5 and 0. Note that this ‘quality control’ is agnostic of the shifting behaviour itself.

We then enquired how much of the variance in the output of the model itself, using parameters comparable to the ones we inferred for the human data, could be explained in a similar manner. If this were comparable, it would indicate that the randomness in the model would be expected to produce a similar uncertainty in predicting preference shift. If it was much greater, it would indicate that additional important processes underpinned variability across participants, not yet described by the model. We thus created a sample of 100 artificial participants whose parameters were sampled randomly from the means +/- 2SD of the inferred parameters. We ran this set of artificial agents with exactly the same task as was given to human participants, and refitted the model to the resulting synthetic data. We found that linearly regressing the preference shift against *σr* , $u$and their interaction explained about 80% of the variance. Unlike Figure 4, the interaction term here explained an extra 10% approx. of useful additional variance.

In the 63.1% of participants who passed the quality criteria above, we found that 84% of the variance in *m_3_*-*m_1_* was captured by regressing this shift against *σr*  , $u_{s}$ and their interaction (cf. fig SF3a vs. SF3b). Thus the amount of variance that the model *might* have explained if it had been precisely correct is large and approximately the same as that actually explained in hyperbolically –discounting participants (84%).

[S4 Fig here]

## We finally fitted in an exploratory manner a slightly more complex model of discounting which might give further insight in the individual variability not captured by the parameters considered so far. We are grateful to our reviewers for this suggestion. We considered a model including a qualitative distinction in evaluating rewards at ‘no delay’ vs. ‘any delay’ that has been made in the literature (e.g. [1,2]) and, for non-zero delays only, we set $V_{D}=C+R_{D}/(1+KD)$. We refer to this as the KTC model. We asked whether: (a) this preference model, including the additive parameter C, described discounting better in our population and (b) if so, whether a change in C accounted for a the change in discounting preferences upon learning to make choices for the partner. To test (a), we fitted this model separately to the self-choices in Phase 1 and Phase 3, and found that the confidence interval for value of C included 0 in about 2/3 of cases (Figure SF5a. and b. below). In addition, the use of the extra parameter was not justified through model comparison (Figure SF5c.). In this comparison, we allowed all parameters of each model, KU or KTC, to vary in each phase separately, thus giving the best possible chance for each model to explain the data but also being agnostic about which parameters might change between phases and why. The result suggested that there was little grounds for adopting the KTC model for describing the population as a whole. There was, however a minority of participants (about 15%) for which BIC values favoured the ‘additive parameter’ model when phase 1 was fitted on its own (Figure SF5c.). We then fitted the KU and KT models for both phases together. The KU model was fitted as before, on the grounds that our Bayesian reasoning suggested that both K and U would change. For the KTC model, however, we fitted a single K and a single T to both phases, while allowing C to vary. This tested the hypothesis that a change in C was responsible for the change in behaviour. We observed that for most (70) participants in the subgroup where the KTC model was the best during the separate fitting, the discounting shift between phases 1 and 3 was also better explained by a change of C (rather than K and U). The KTC model was not specifically tailored to test the Bayesian-shift hypothesis but we can still ask whether it provides evidence for or against the Bayesian prediction that shifting should correlate with decision variability. We tested this for the 70 participants whose shifting was best explained as a change in C and found powerful evidence in favour of the Bayesian prediction (Fig SF5.).

[S5 Fig here]

**References to the Supplementary Information**

1. Angeletos G-M, Laibson D, Repetto A, Tobacman J, Weinberg S. The hyperbolic consumption model: Calibration, simulation, and empirical evaluation. Journal of Economic Perspectives. JSTOR; 2001;47–68.

2. McClure SM, Laibson DI, Loewenstein G, Cohen JD. Separate neural systems value immediate and delayed monetary rewards. Science. American Association for the Advancement of Science; 2004;306(5695):503–507.
