# Supplementary figures and images for: How People Use Social Information to Find out What to Want in the Paradigmatic Case of Inter-temporal Preferences
Source: PLoS Comput Biol. 2016 Jul 22;12(7):e1004965. doi: 10.1371/journal.pcbi.1004965 (PMC4957786; doi:10.1371/journal.pcbi.1004965)

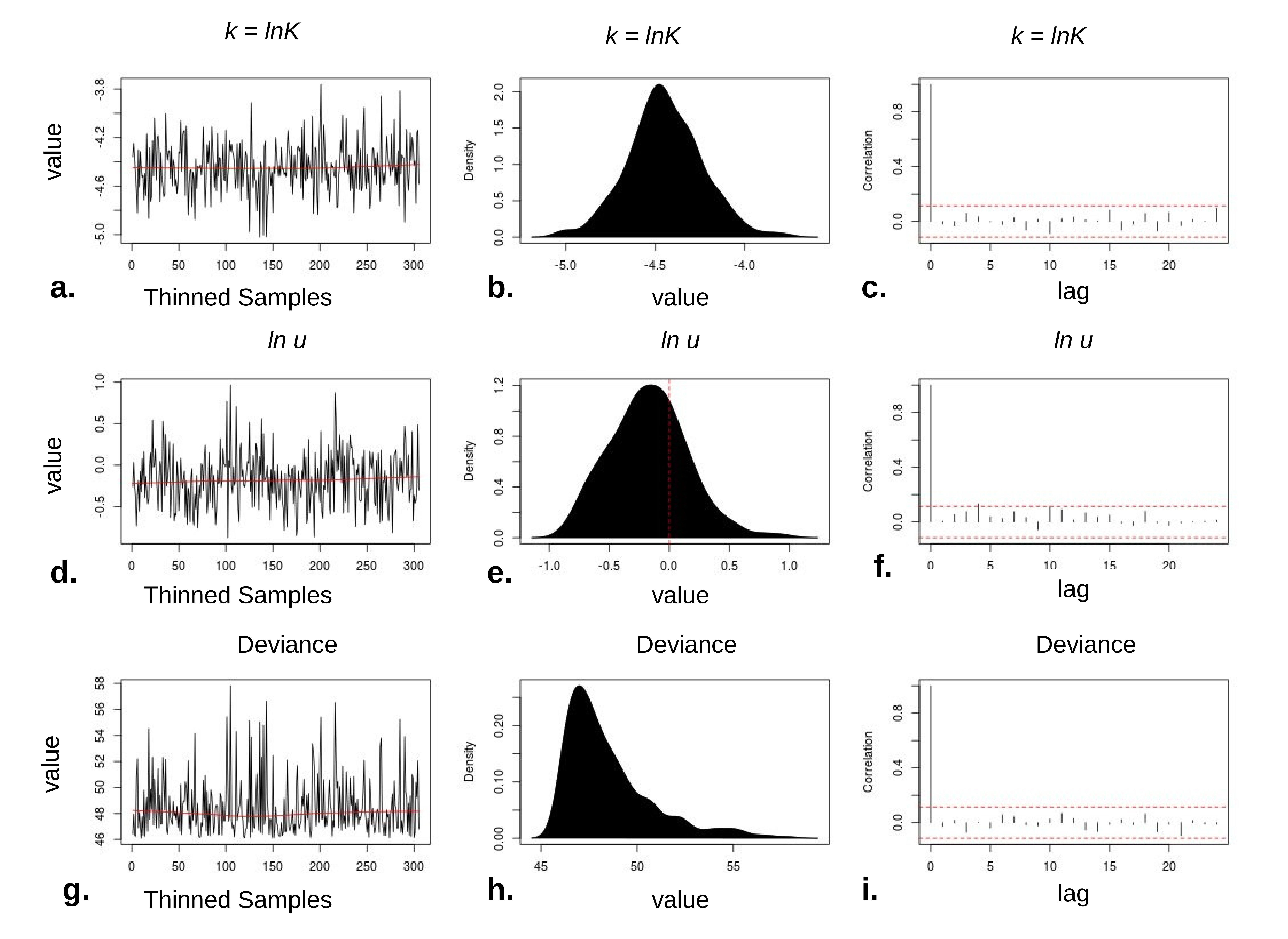

Supplement: S1 Fig — The fit was parametrised as per the KU model, using only trials from the phase 1 of the experiment, before exposure to the choices of another agent. A participant with fitted values near the middle of the population distribution of Fig 2 is shown. The three rows of plots represent the values of the (log) preference parameter, the (log) uncertainty parameter and the model deviance–a measure of model fit derived from the log-likelihood—as more samples are obtained from the posterior distribution of the parameters (the converged Markov Chain). The three columns show: a., d., g.: Values at consecutive thinned samples, illustrating that stability has been achieved. b., e., h.: smoothed histograms representing the posterior distributions. Note that they have very well defined peaks both for k and for u. c., f., i.: Autocorrelation plots indicating that the degree of thinning was appropriate–i.e., that consecutive samples (from the first column) used to construct the posteriors (second column) were independent. (TIFF) [file pcbi.1004965.s003.tiff]

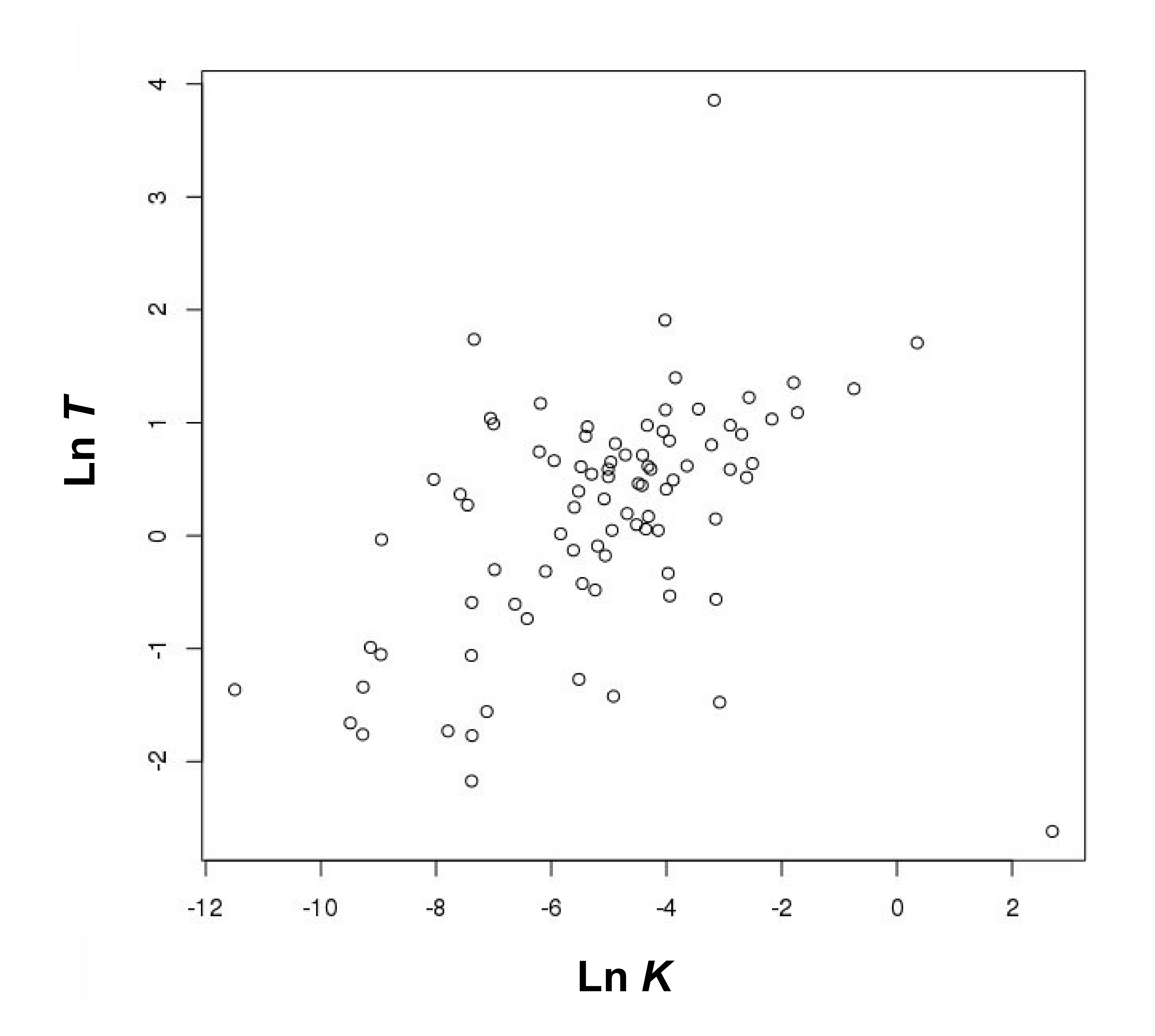

Supplement: S2 Fig — This is analogous to Fig 2A but produced by applying the experimental task to artificial agents. These agents followed the KU model with uncorrelated km and ku. (TIFF) [file pcbi.1004965.s004.tiff]

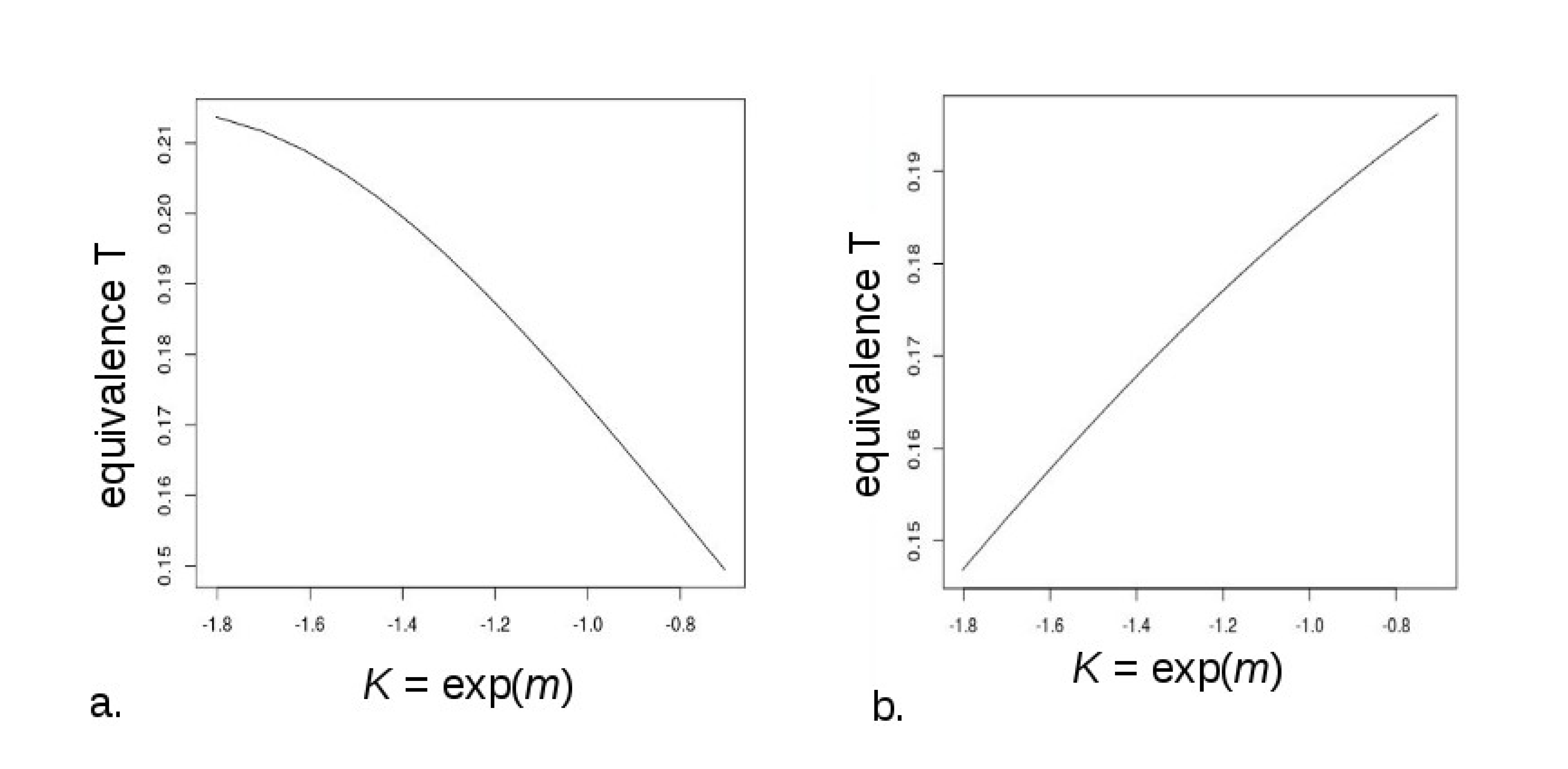

Supplement: S3 Fig — The plots show the temperature parameter that an agent with the same modal discounting preference, but following the classic KT model, has to have in order to display an indistinguishable policy. a. The same option pair, Ro = 1 vs. Rd = 3, D = 10 is presented to all agents. b. The same Ro and D are used as in a., but Rd gradually increases from 2 to 4. This results in the indifference point between the options being 1 x u below m, but this is not important as long as kind tracks m. (TIFF) [file pcbi.1004965.s005.tiff]

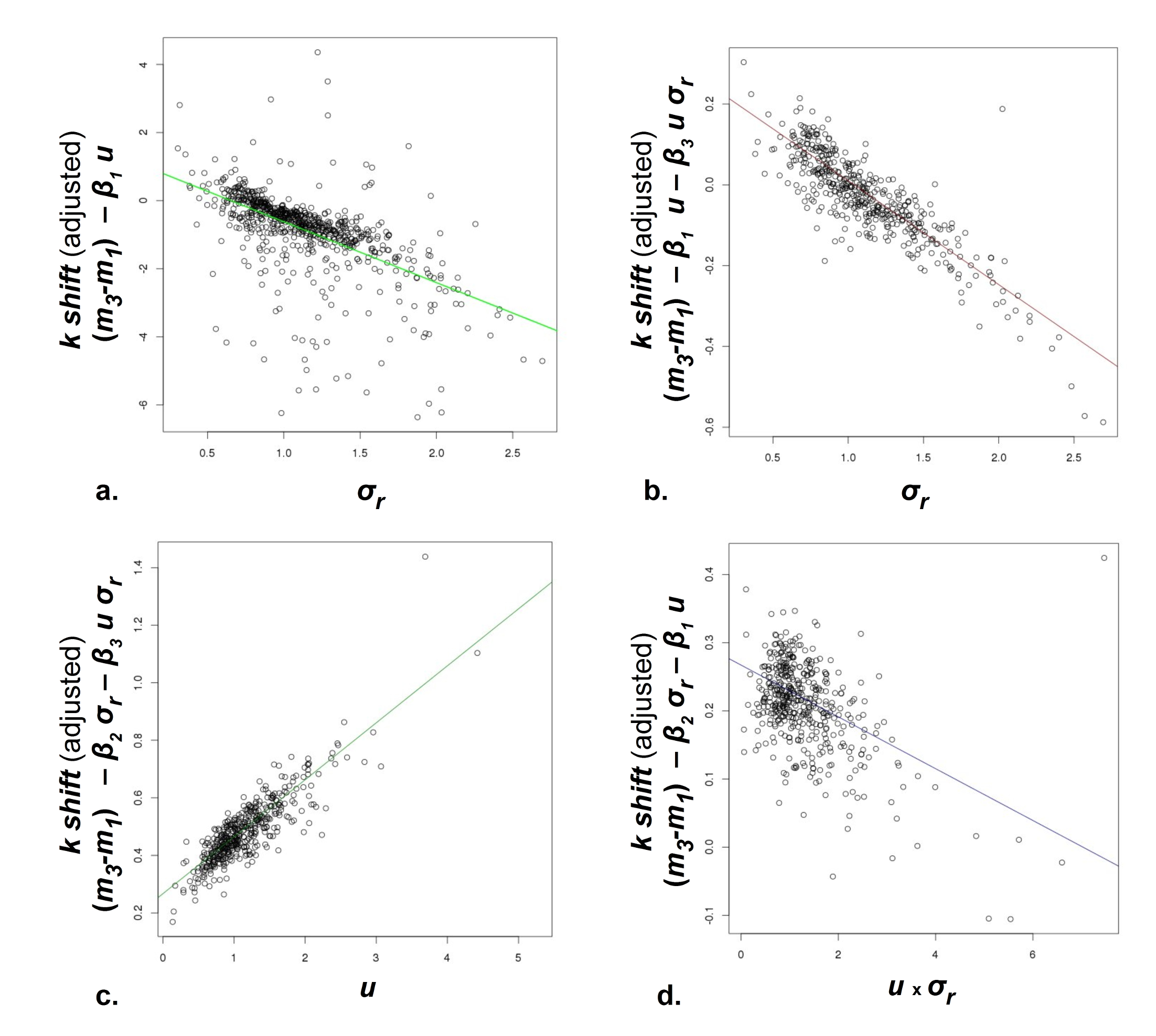

Supplement: S4 Fig — This is based on fitting a linear regression m3 –m1 = β0 + β1 u +β2 σr + β3 u σr to the ‘quality-contolled’ data set only. In all plots m3-m1 is considered positive if towards the preferences of the Other, and negative in the opposite direction. a. Shift magnitude vs. reference population dispersion σr in the entire population. Grey: resulting regression line according to the ‘quality controlled’ dataset. b. Similar plot restricted to the ‘quality-controlled’ dataset. This picks out the area of high correlation in a. and excludes its penumbra. β0 to β3 are derived from this set, N = 466. c. preference variability is also tightly related to shift in this set, while d. the u σr interaction also makes a contribution, as in the simulated data. p for all β is < 1e-16. (TIFF) [file pcbi.1004965.s006.tiff]

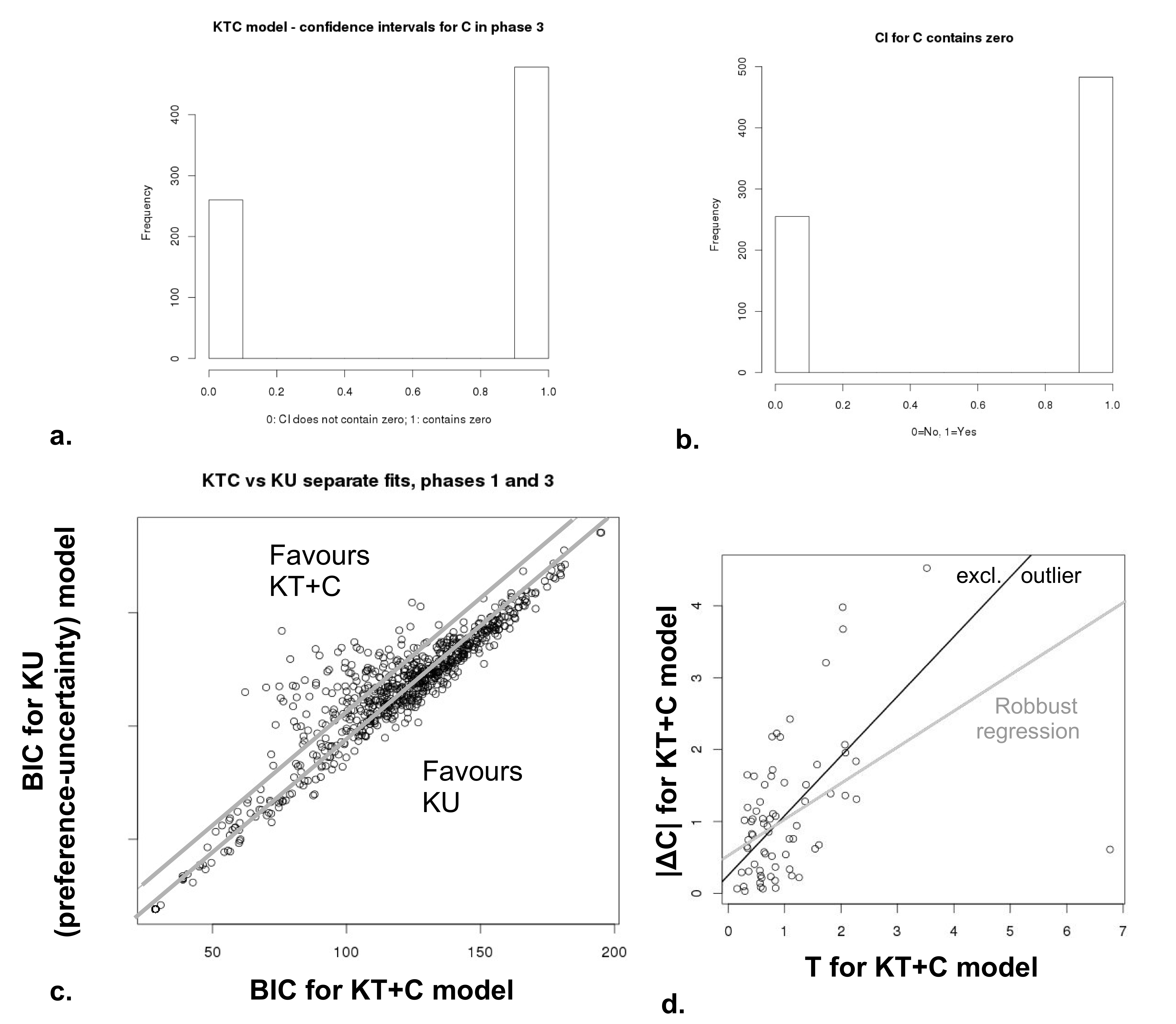

Supplement: S5 Fig — a. and b. For about 2/3 of participants in both phases 1 and 3 the confidence interval for C includes zero (‘KTC model’: the value of the delayed option is adjusted by and individual parameter C). c. Comparison of BIC values for KU model vs. KTC model. The two grey lines indicated +/- 6 BIC units, conventionally taken to be ‘strong evidence’. Many more points are below these lines than above (403 vs. 108; ΔBIC = 744.9 over 648 participants in favour of KU). The KU model gives a better account of behaviour over the whole group, but there is a tail of participants where the KT+C model fits better. For most of these participants preference shifting is also better described as a change in C. d. Relationship between decision variability and preference shift for the 70 participants whose preference shift was best fitted by a change in C according to the KTC model. There is a very strong correlation between decision variability and shifting, as a Bayesian update would predict (r = 0.39, p = 0.00048 overall; r = 0.64, p = 3e-12 excluding the single outlier). (TIFF) [file pcbi.1004965.s007.tiff]
